# Supplementary figures and images for: Genetic Analysis of Avian Coronavirus Infectious Bronchitis Virus in Yellow Chickens in Southern China over the Past Decade: Revealing the Changes of Genetic Diversity, Dominant Genotypes, and Selection Pressure
Source: Viruses. 2019 Sep 26;11(10):898. doi: 10.3390/v11100898 (PMC6833030; doi:10.3390/v11100898)

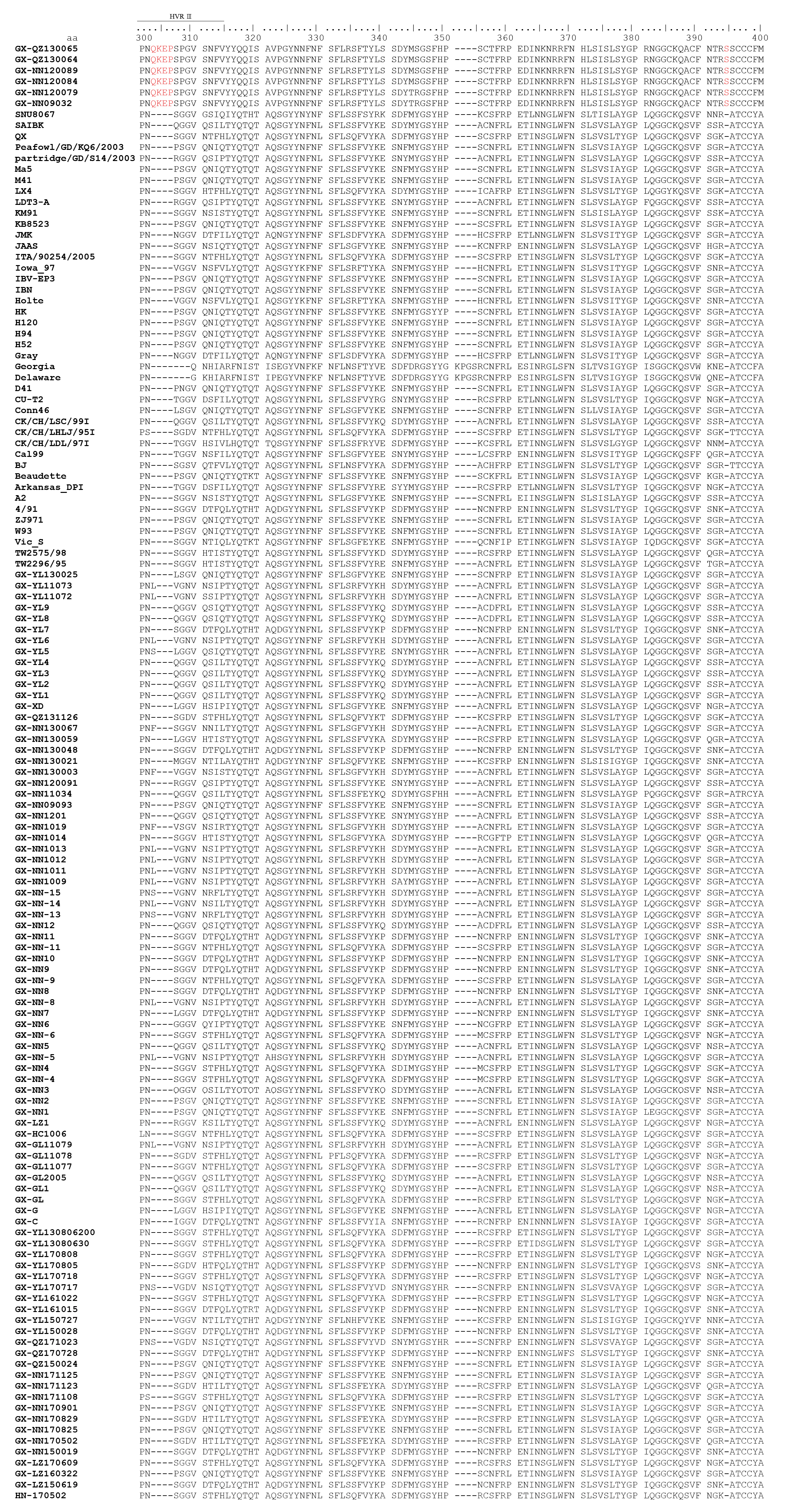

Supplement: Supplementary file 1 [file viruses-11-00898-s001.zip › Supplementary Figure S1.jpg]

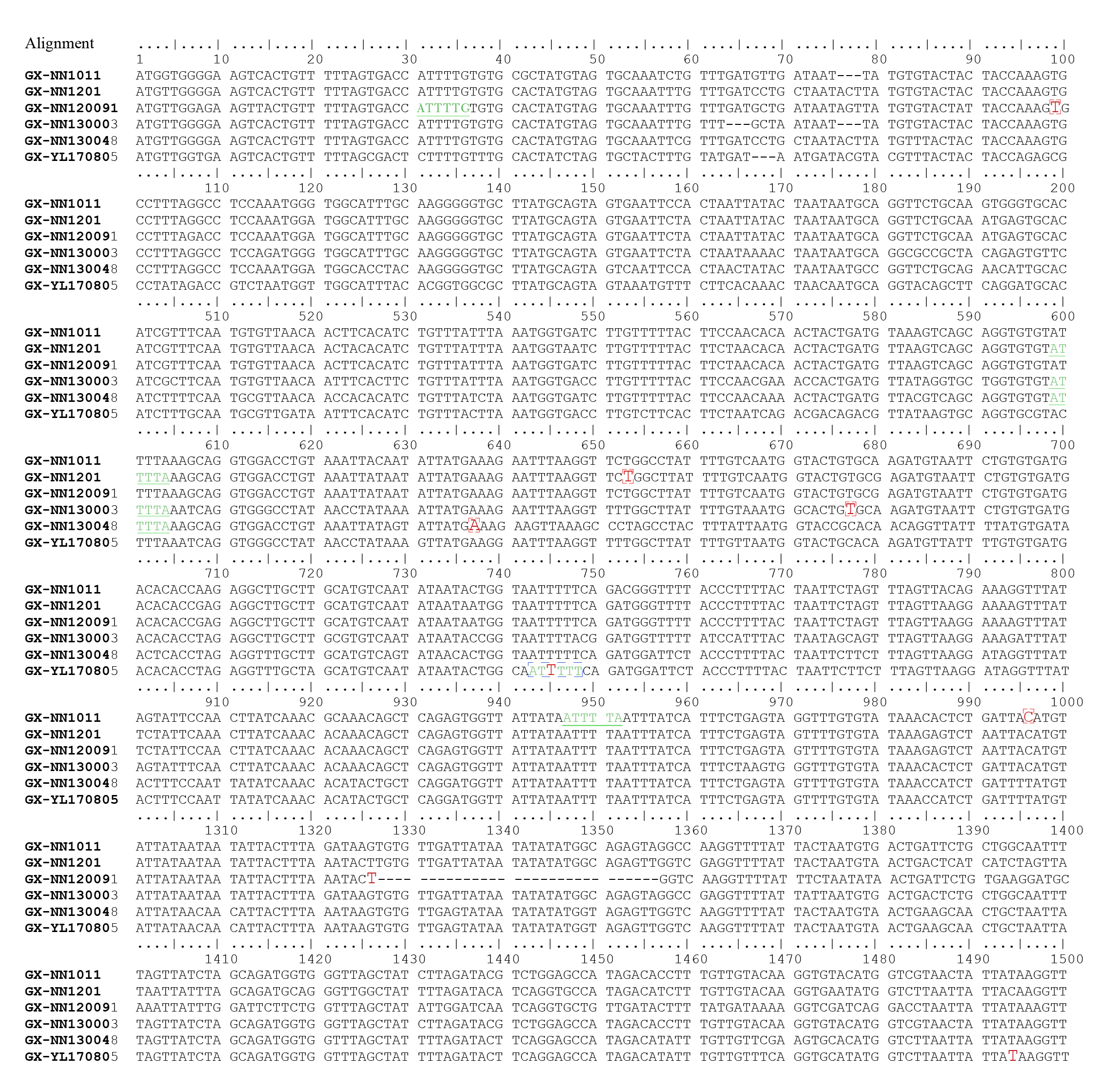

Supplement: Supplementary file 1 [file viruses-11-00898-s001.zip › Supplementary Figure S2.jpg]

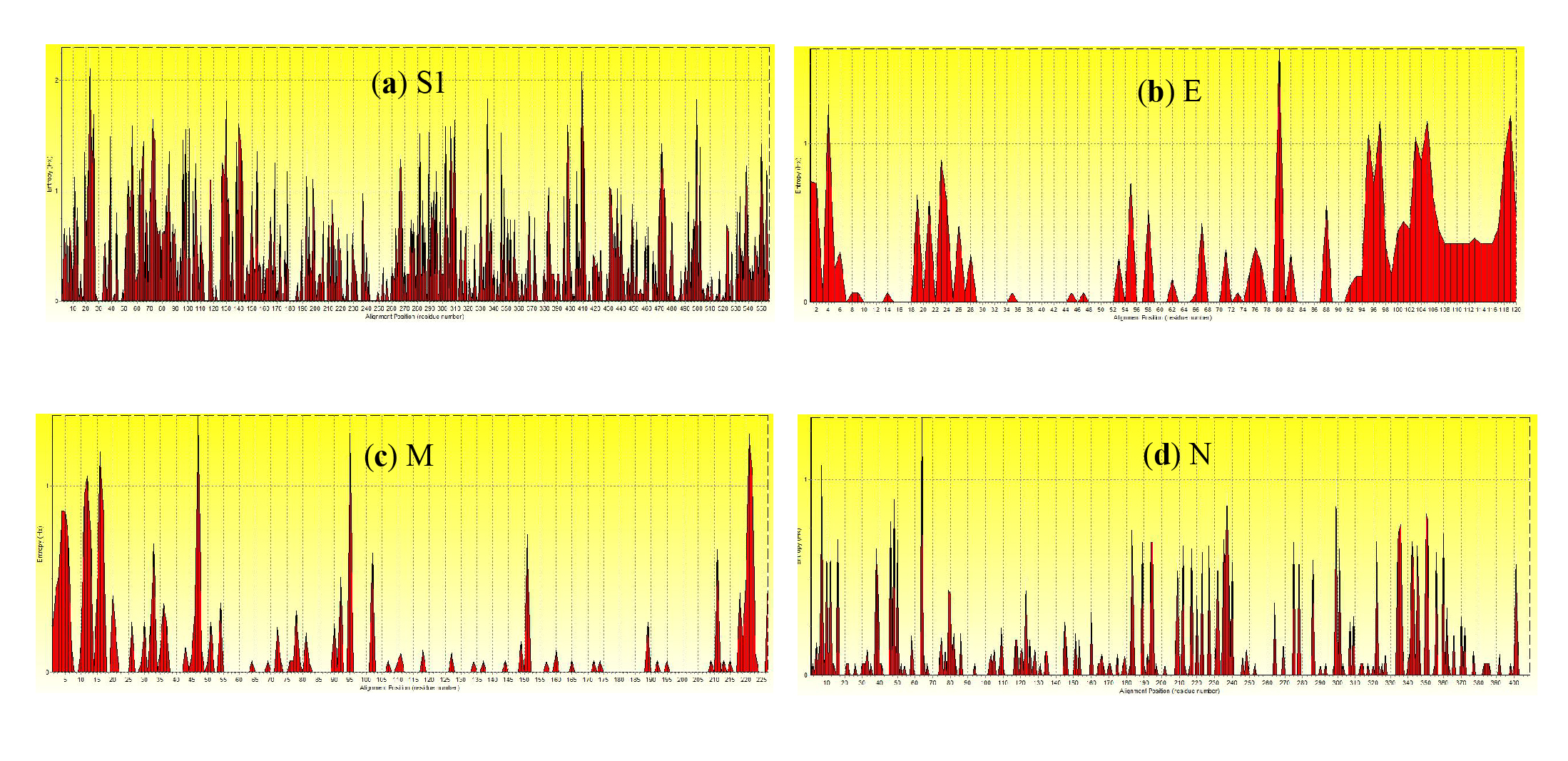

Supplement: Supplementary file 1 [file viruses-11-00898-s001.zip › Supplementary Figure S3.jpg]

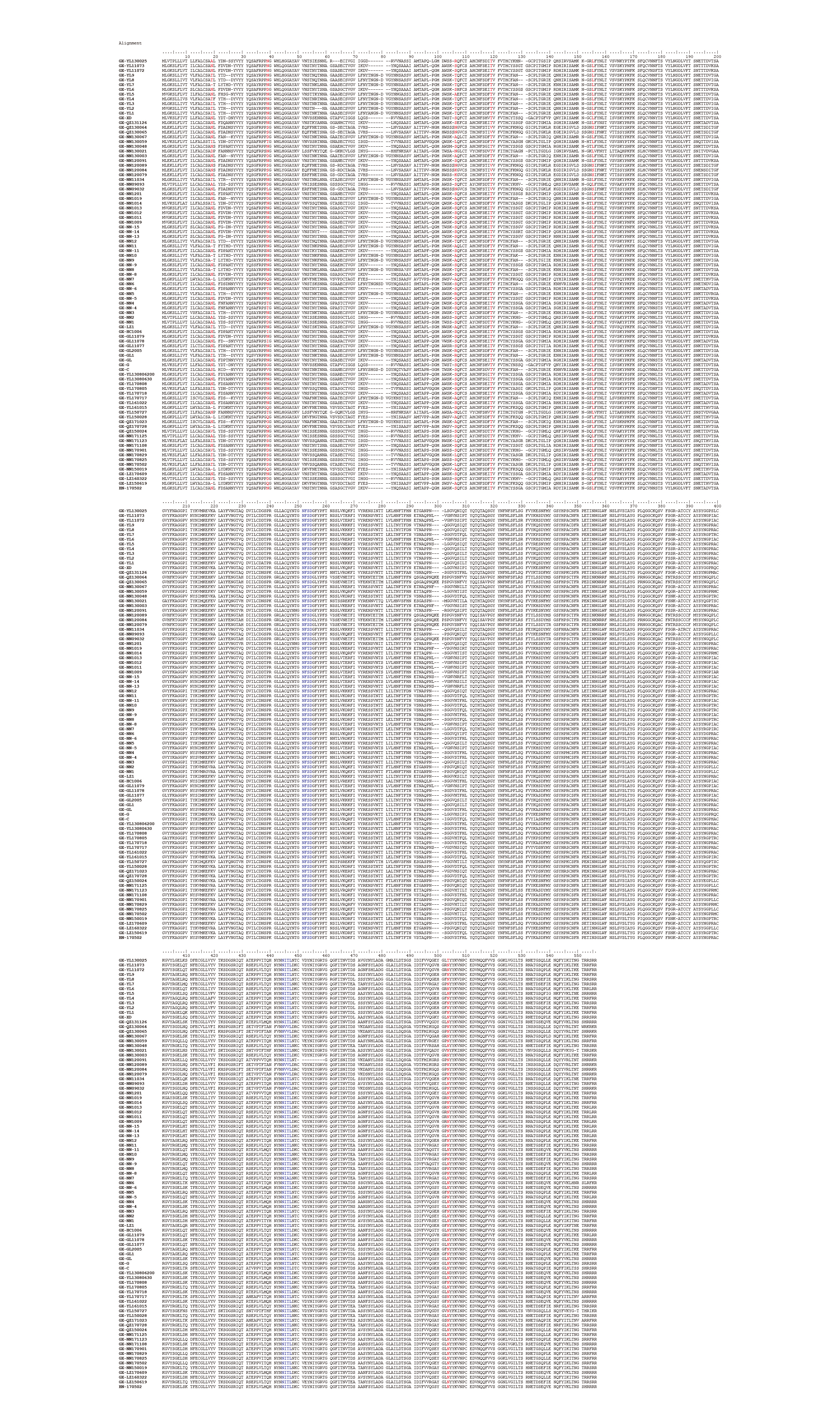

Supplement: Supplementary file 1 [file viruses-11-00898-s001.zip › Supplementary Figure S4.jpg]

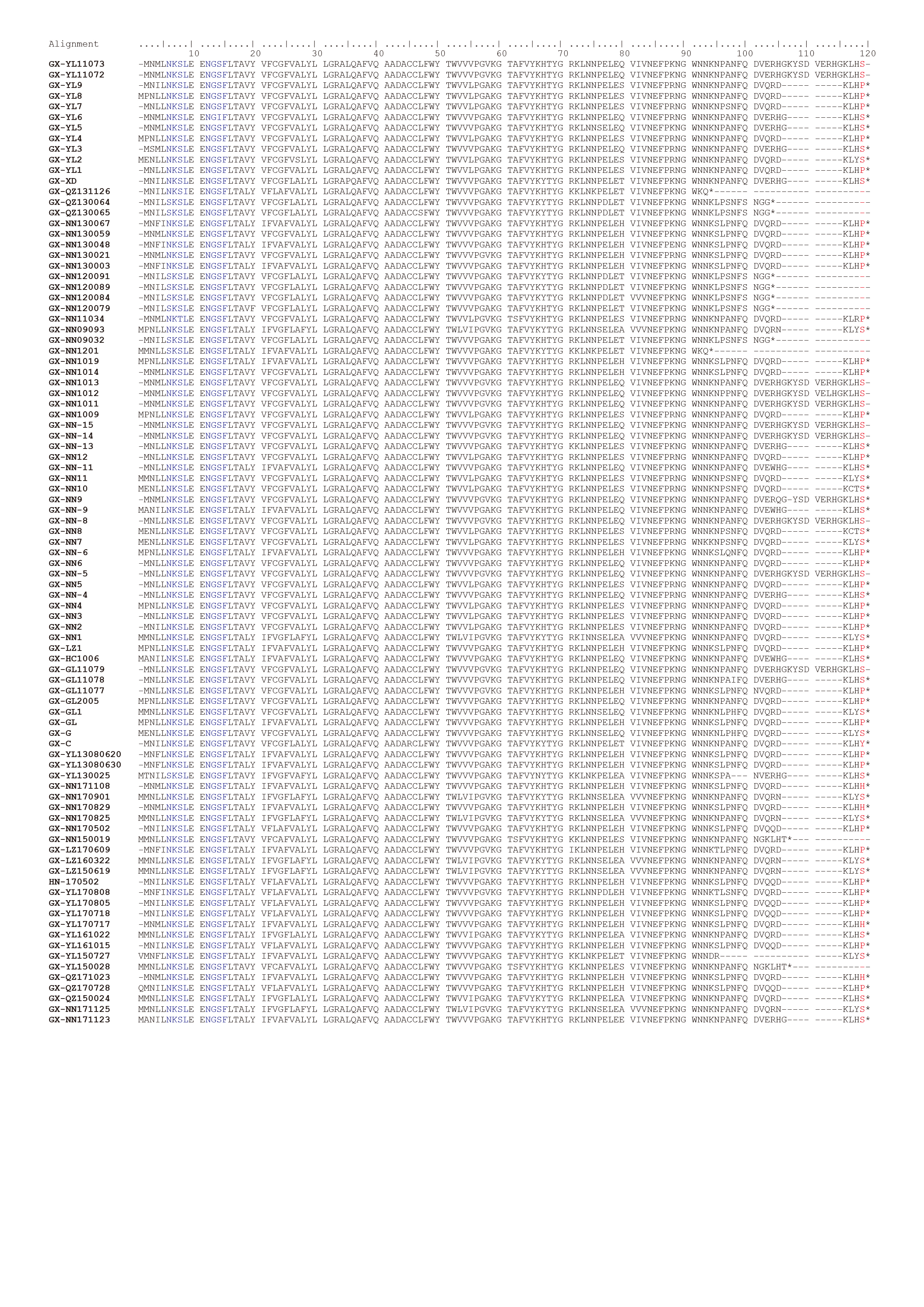

Supplement: Supplementary file 1 [file viruses-11-00898-s001.zip › Supplementary Figure S5.jpg]

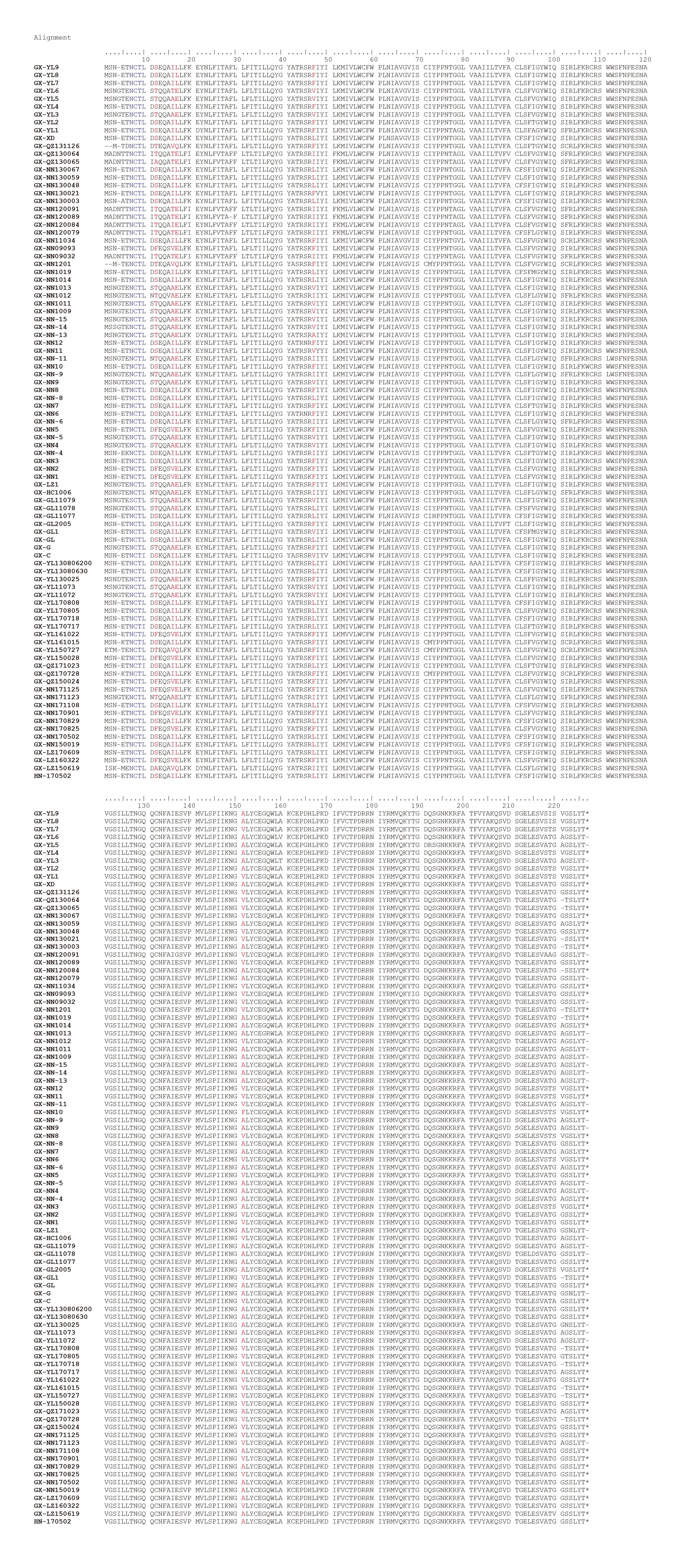

Supplement: Supplementary file 1 [file viruses-11-00898-s001.zip › Supplementary Figure S6.jpg]

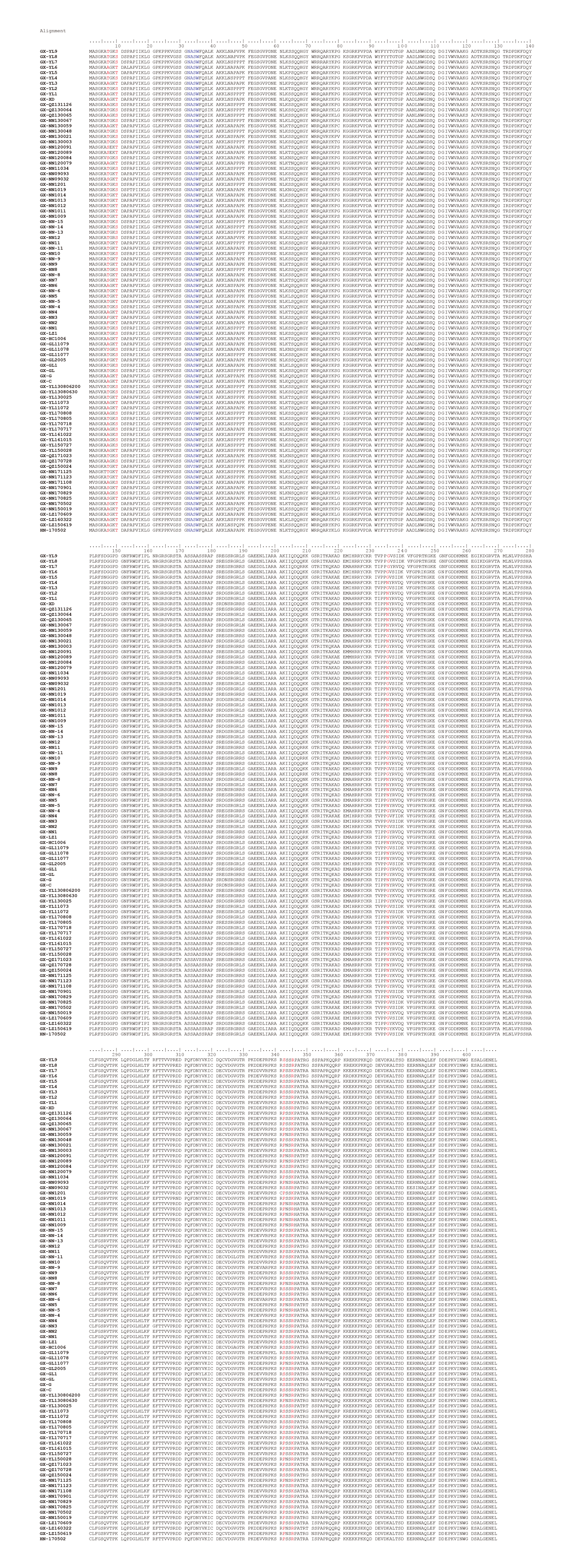

Supplement: Supplementary file 1 [file viruses-11-00898-s001.zip › Supplementary Figure S7.jpg]
