# Supplementary material for: Genetic Analysis of Avian Coronavirus Infectious Bronchitis Virus in Yellow Chickens in Southern China over the Past Decade: Revealing the Changes of Genetic Diversity, Dominant Genotypes, and Selection Pressure
Source: Viruses. 2019 Sep 26;11(10):898. doi: 10.3390/v11100898 (PMC6833030; doi:10.3390/v11100898)
Supplement: Supplementary file 1 [file viruses-11-00898-s001.zip › Supplementary Table S1.doc]

**Supplementary Table S1.** IBV reference strains used in this study.

| **IBV strains** | **Years of isolation** | **pathogenesis** | **Country of origin** | **Accession numbers** |
| --- | --- | --- | --- | --- |
| M41 | Vaccine | Respiratory | USA | GQ504725 (S1, M, N, E)^a^ |
| H120 | Vaccine | Respiratory | Netherlands | GU393335 (S1, M, N, E) |
| H52 | Vaccine | Respiratory | Netherlands | EU817497 (S1, M, N, E) |
| Ma5 | Vaccine | Nephropathogenic | USA | AY561713 (S1)  GQ174486 (M)  HQ014604 (N)  KY626045 (E) |
| 4/91 | Vaccine | Respiratory | UK | AF093793 (S1)  GQ227602 (M)  EU780081 (N)  KF377577 (E) |
| W93 | Vaccine | Nephropathogenic | China | AY842862 (S1)  EF602443 (M, N, E) |
| H94 | Vaccine | NA^b^ | China | DQ515801 (S1)  EF602438 (M, N, E) |
| D41 | Vaccine | NA | China | AF036937 (S1)  EF602437 (M, N, E) |
| IBN | Vaccine | NA | China | AY856348 (S1)  EF602439 (M, N, E) |
| HK | Vaccine | Nephropathogenic | China | AY761141 (S1, M, N, E) |
| JAAS | Vaccine | NA | China | AY839140 (S1)  EF602441 (M, N, E) |
| Arkansas DPI | 1981 | Respiratory | USA | GQ504720 (S1, M, N, E) |
| Beaudette | 1937 | Respiratory | USA | NC_001451 (S1, M, N, E) |
| Conn46 1972 | 1972 | NA | USA | FJ904717 (S1, M, N, E) |
| Delaware 072 | 1992 | NA | USA | GU393332 (S1, M, N, E) |
| Cal99 | 1999 | NA | USA | AY514485 (S1, M, N, E) |
| Gray | 1960 | Nephropathogenic | USA | GU393334 (S1, M, N, E) |
| Georgia | 1998 | NA | USA | GQ504722 (S1, M, N, E) |
| Iowa | 1956 | NA | USA | GU393337 (S1, M, N, E) |
| JMK | 1964 | NA | USA | GU393338 (S1, M, N, E) |
| Holte | 1954 | Nephropathogenic | USA | GU393336 (S1, M, N, E) |
| CU-T2 | 1995 | Respiratory variant | USA | U49858 (S1, M, N, E) |
| Vic S | Vaccine | Nephropathogenic | Australia | DQ490221 (S1, M, N, E) |
| ITA/90254/2005 | 2005 | NA | Western Africa | FN430414 (S1, M, N, E) |
| IBV-EP3 | NA | NA | Singapore | DQ001338 (S1, M, N, E) |
| KB8523 | 1988 | Nephropathogenic | Japan | M21515 (S1, M, N, E) |
| TW2296/95 (II) | 1995 | Nephropathogenic | Taiwan | DQ646404 (S1, M, N, E) |
| TW2575/98 (I) | 1998 | Nephropathogenic | Taiwan | DQ646405 (S1, M, N, E) |
| LX4 | 1999 | Nephropathogenic | China | AY338732 (S1, M, N, E) |
| KM91 | 1991 | Nephropathogenic | Korea | JQ977698 (S1, M, N, E) |
| SNU8067 | 2008 | Respiratory | Korea | JQ977697 (S1, M, N, E) |
| QX | 1997 | Proventriculus | China | AF193423 (S1)  AF221667 (M)  AF199412 (N) |
| A2 | 1996 | Nephropathogenic | China | EU526388 (S1, M, N, E) |
| ZJ971 | 1997 | Proventriculus | China | EU714028 (S1, M, N, E) |
| BJ | 1997 | Nephropathogenic | China | AY319651 (S1, M, N, E) |
| SAIBK | NA | Nephropathogenic | China | DQ288927 (S1, M, N, E) |
| LDT3 | 2003 | Nephropathogenic | China | AY702975 (S1, M, N, E) |
| CK/CH/LSC/99I | 1999 | NA | China | DQ167147 (S1)  EF602450 (M, N, E) |
| CK/CH/LDL/97I | 1997 | NA | China | EF030997 (S1)  EF602445 (M, N, E) |
| CK/CH/LHLJ/95I | 1995 | NA | China | DQ167141 (S1)  EF602454 (M, N, E) |
| Peafowl/GD/KQ6/2003 | 2003 | NA | China | AY641576 (S1, M, N, E) |
| Partridge/GD/S14/2003 | 2003 | NA | China | AY646283 (S1, M, N, E) |

^a^ Sequences of the gene fragments used within the parenthesis.

^b^ NA: data not available
