# Supplementary material for: Genetic Analysis of Avian Coronavirus Infectious Bronchitis Virus in Yellow Chickens in Southern China over the Past Decade: Revealing the Changes of Genetic Diversity, Dominant Genotypes, and Selection Pressure
Source: Viruses. 2019 Sep 26;11(10):898. doi: 10.3390/v11100898 (PMC6833030; doi:10.3390/v11100898)
Supplement: Supplementary file 1 [file viruses-11-00898-s001.zip › Supplementary Table S2.doc]

**Supplementary Table S2.** Estimates of evolutionary distances over sequence pairs between groups of S1 gene.

| **Gene type** | **No. of base substitutions per site in lower triangle or standard error in upper triangle ^a^** | | | | | | | | |
| --- | --- | --- | --- | --- | --- | --- | --- | --- | --- |
|  | **LX4** | **CK/CH/LSC/99I** | **LDT3-A** | **4/91** | **Taiwan-I** | **Taiwan-Ⅱ** | **Mass** | **New-type 2** | **New-type 1** |
| LX4 |  | 0.016 | 0.013 | 0.015 | 0.016 | 0.016 | 0.017 | 0.019 | 0.020 |
| CK/CH/LSC/99I | 0.209 |  | 0.013 | 0.013 | 0.015 | 0.015 | 0.017 | 0.019 | 0.021 |
| LDT3-A | 0.152 | 0.155 |  | 0.014 | 0.014 | 0.014 | 0.016 | 0.018 | 0.019 |
| 4/91 | 0.200 | 0.175 | 0.229 |  | 0.016 | 0.016 | 0.017 | 0.018 | 0.020 |
| Taiwan-I | 0.199 | 0.162 | 0.188 | 0.224 |  | 0.013 | 0.016 | 0.019 | 0.020 |
| Taiwan-Ⅱ | 0.222 | 0.171 | 0.194 | 0.226 | 0.144 |  | 0.015 | 0.019 | 0.020 |
| Mass | 0.222 | 0.196 | 0.202 | 0.242 | 0.169 | 0.156 |  | 0.019 | 0.020 |
| New-type 2 | 0.339 | 0.342 | 0.337 | 0.341 | 0.341 | 0.335 | 0.327 |  | 0.021 |
| New-type 1 | 0.389 | 0.415 | 0.384 | 0.414 | 0.404 | 0.421 | 0.412 | 0.408 |  |

^a^ All results are based on the pairwise analysis of 91 isolates sequences during 1985-2017 and the number of sequences analyzed per groups was: LX4-type, n=20; CK/CH/LSC/99I-type, n=19; LDT3-A-type, n=15; 4/91-type, n=13; Taiwan-I-type, n=5; Taiwan-Ⅱ-type, n=2; Mass-type, n=9; New-type 2-type, n=2; New-type 1-type, n=6. Analyses were conducted using the Maximum Composite Likelihood model by a bootstrap procedure (1000 replicates). All positions containing gaps and missing data were eliminated. There were a total of 502 positions in the final dataset. Evolutionary analyses were conducted in MEGA 6.06.
