# Supplementary material for: Genetic Analysis of Avian Coronavirus Infectious Bronchitis Virus in Yellow Chickens in Southern China over the Past Decade: Revealing the Changes of Genetic Diversity, Dominant Genotypes, and Selection Pressure
Source: Viruses. 2019 Sep 26;11(10):898. doi: 10.3390/v11100898 (PMC6833030; doi:10.3390/v11100898)
Supplement: Supplementary file 1 [file viruses-11-00898-s001.zip › Supplementary Table S3.doc]

**Supplementary Table S3.** Estimates of evolutionary distances over sequence pairs between groups of E gene.

| **Gene type** | **No. of base substitutions per site in lower triangle or standard error in upper triangle ^a^** | | | | | |
| --- | --- | --- | --- | --- | --- | --- |
|  | **CK/CH/LSC/99I** | **4/91** | **LDT3-A** | **LX4** | **Mass** | **Conn** |
| CK/CH/LSC/99I |  | 0.129 | 0.022 | 0.024 | 0.034 | 0.028 |
| 4/91 | 0.060 |  | 0.023 | 0.025 | 0.037 | 0.026 |
| LDT3-A | 0.071 | 0.065 |  | 0.018 | 0.034 | 0.027 |
| LX4 | 0.091 | 0.090 | 0.051 |  | 0.032 | 0.026 |
| Mass | 0.147 | 0.158 | 0.141 | 0.123 |  | 0.033 |
| Conn | 0.127 | 0.106 | 0.106 | 0.100 | 0.147 |  |

^a^ All results are based on the pairwise analysis of 91 isolates sequences during 1985-2017 and the number of sequences analyzed per groups was: CK/CH/LSC/99I-type, n=50; 4/91-type, n=3; LDT3-A-type, n=7; LX4-type, n=18; Mass-type, n=9; Conn-type, n=4. Analyses were conducted using the Maximum Composite Likelihood model by a bootstrap procedure (1000 replicates). All positions containing gaps and missing data were eliminated. There were a total of 92 positions in the final dataset. Evolutionary analyses were conducted in MEGA 6.06.
