# Supplementary material for: Genetic Analysis of Avian Coronavirus Infectious Bronchitis Virus in Yellow Chickens in Southern China over the Past Decade: Revealing the Changes of Genetic Diversity, Dominant Genotypes, and Selection Pressure
Source: Viruses. 2019 Sep 26;11(10):898. doi: 10.3390/v11100898 (PMC6833030; doi:10.3390/v11100898)
Supplement: Supplementary file 1 [file viruses-11-00898-s001.zip › Supplementary Table S4.doc]

**Supplementary Table S4.** Estimates of evolutionary distances over sequence pairs between groups of M gene.

| **Gene type** | **No. of base substitutions per site in lower triangle or standard error in upper triangle ^a^** | | | |
| --- | --- | --- | --- | --- |
|  | **CK/CH/LSC/99I** | **QX** | **Mass** | **4/91** |
| CK/CH/LSC/99I |  | 0.015 | 0.013 | 0.013 |
| QX | 0.079 |  | 0.015 | 0.013 |
| Mass | 0.059 | 0.079 |  | 0.013 |
| 4/91 | 0.060 | 0.069 | 0.055 |  |

^a^ All results are based on the pairwise analysis of 91 isolates sequences during 1985-2017 and the number of sequences analyzed per groups was: CK/CH/LSC/99I-type, n=45; QX-type, n=31; Mass-type, n=11; 4/91-type, n=4. Analyses were conducted using the Maximum Composite Likelihood model by a bootstrap procedure (1000 replicates). All positions containing gaps and missing data were eliminated. There were a total of 221 positions in the final dataset. Evolutionary analyses were conducted in MEGA 6.06.
