# Supplementary material for: Genetic Analysis of Avian Coronavirus Infectious Bronchitis Virus in Yellow Chickens in Southern China over the Past Decade: Revealing the Changes of Genetic Diversity, Dominant Genotypes, and Selection Pressure
Source: Viruses. 2019 Sep 26;11(10):898. doi: 10.3390/v11100898 (PMC6833030; doi:10.3390/v11100898)
Supplement: Supplementary file 1 [file viruses-11-00898-s001.zip › Supplementary Table S5.doc]

**Supplementary Table S5.** Estimates of evolutionary distances over sequence pairs between groups of N gene.

| **Gene type** | **No. of base substitutions per site in lower triangle or standard error in upper triangle ^a^** | | | | | |
| --- | --- | --- | --- | --- | --- | --- |
|  | **LX4** | **CK/CH/LSC/99I** | **New** | **4/91** | **Peafowl/GD/KQ** | **Mass** |
| LX4 |  | 0.010 | 0.009 | 0.010 | 0.009 | 0.010 |
| CK/CH/LSC/99I | 0.082 |  | 0.013 | 0.011 | 0.011 | 0.012 |
| New | 0.071 | 0.088 |  | 0.011 | 0.010 | 0.011 |
| 4/91 | 0.078 | 0.079 | 0.069 |  | 0.009 | 0.009 |
| Peafowl/GD/KQ | 0.071 | 0.071 | 0.056 | 0.052 |  | 0.008 |
| Mass | 0.084 | 0.085 | 0.068 | 0.051 | 0.042 |  |

^a^ All results are based on the pairwise analysis of 91 isolates sequences during 1985-2017 and the number of sequences analyzed per groups was: LX4-type, n=54; CK/CH/LSC/99I-type, n=22; New-type, n=4; 4/91-type, n=4; Peafowl/GD/KQ -type, n=1; Mass-type, n=6. Analyses were conducted using the Maximum Composite Likelihood model by a bootstrap procedure (1000 replicates). All positions containing gaps and missing data were eliminated. There were a total of 409 positions in the final dataset. Evolutionary analyses were conducted in MEGA 6.06.
