# Supplementary material for: Genetic Analysis of Avian Coronavirus Infectious Bronchitis Virus in Yellow Chickens in Southern China over the Past Decade: Revealing the Changes of Genetic Diversity, Dominant Genotypes, and Selection Pressure
Source: Viruses. 2019 Sep 26;11(10):898. doi: 10.3390/v11100898 (PMC6833030; doi:10.3390/v11100898)
Supplement: Supplementary file 1 [file viruses-11-00898-s001.zip › Supplementary Table S6.doc]

**Supplementary Table S6.** The selection profile and entropy values of S1, E, M and N proteins of Guangxi IBV isolates.

| **Protein** | **Position sites** | **Entropy values** |
| --- | --- | --- |
| S1 | 19 | 1.29740 |
|  | 39 | 1.56670 |
|  | 106 | 1.39276 |
|  | 118 | 1.11306 |
|  | 154 | 1.36213 |
|  | 503 | 1.39069 |
| E | 119 | 1.17114 |
| M | 12 | 1.05539 |
|  | 16 | 1.18695 |
|  | 17 | 0.84243 |
|  | 47 | 1.37949 |
|  | 151 | 0.73991 |
| N | 7 | 1.07497 |
|  | 10 | 0.69019 |
|  | 235 | 0.69309 |
|  | 342 | 0.68212 |
|  | 345 | 0.66086 |
